# Hot Deformation Behavior and Strain-Compensated Constitutive Equation of Nano-Sized SiC Particle-Reinforced Al-Si Matrix Composites

**DOI:** 10.3390/ma13081812

**Published:** 2020-04-11

**Authors:** Zhen Wang, Aiqin Wang, Jingpei Xie, Pei Liu

**Affiliations:** 1 School of Materials Science and Engineering, Henan University of Science and Technology, Luoyang 471023, China; 2 Collaborative Innovation Center of Non-Ferrous Materials of Henan Province, Luoyang 471023, China

**Keywords:** nano-SiCp/Al-Si composites, hot deformation behavior, strain compensation, activation energy

## Abstract

The hot deformation behavior of nano-SiCp/Al-Si composites was studied by isothermal compression tests at 470–530 °C and strain rates of 0.01–5 s^−1^. A strain-compensation constitutive model was developed with a Z parameter and an Arrhenius function, and its accuracy was verified by error analysis. The results show that the flow stress of the composites decreased with the increase in deformation temperature and the decrease in strain rate. The average activation energy for nano-SiC particle-reinforced Al-Si matrix composites was 277 kJ/mol, which was larger than the activation energy for self-diffusion of pure aluminum. The average relative error was calculated as 2.88%, indicating the strain-compensated constitutive equation could accurately predict the hot deformation behavior of nano-SiCp/Al-Si composites.

## 1. Introduction

SiCp/Al composites have attracted significant attention in applications for rail transit, aerospace, the automotive industry, and electronic packaging because of their excellent properties [1,2,3,4]. Traditionally, SiCp/Al composites are usually fabricated using micro-sized SiC particles. However, the addition of micro-sized SiC particles to an aluminum alloy can greatly reduce the plasticity of the material while improving strength, which limits the applicability of micro-sized SiC particle-reinforced aluminum matrix composites in the industrial field [5,6,7]. In recent years, researchers have found that the introduction of nano-sized particles to an aluminum alloy can greatly improve the hardness and ultimate tensile strength under the premise of retaining plasticity [8,9,10,11]. For instance, Zhang et al. [8] found that adding 0.5% nano-SiC particles to 2014 alloy improved the tensile strength of Al2014 alloy from 240 MPa to 314 MPa at 493 K while maintaining ductility. Mousavian et al. [11] studied the tensile strength and fracture strain of nano-SiCp/A356 composites prepared by casting, and found that the yield strength, fracture strain, and the ultimate tensile strength of the nano-SiCp/A356 composites were 85%, 70%, and 77% higher than that of unreinforced aluminum alloy, respectively.

It is well known that SiCp/Al composites usually require secondary deformation, such as forging and hot rolling, before being employed in the industrial field because the properties of the composites can be significantly improved by hot deformation. In recent years, many scholars have performed numerous studies on the hot deformation behavior of a number of alloys and composites and developed corresponding constitutive equations [12,13,14,15,16,17]. For instance, Chen et al. [12] constructed a constitutive equation for hybrid particle-reinforced aluminum matrix composites and found an activation energy of the composites of 269 kJ/mol. Gangolu et al. [16] found that the instability domain of the A356 alloy changed from 510–570 °C at a strain rate of 0.01–0.04 s^−1^ to 470–570 °C at the same strain rate with the addition of 5% B_4_C particles. All of the above results indicated that the investigation of deformation behavior and constitutive equations could lay a foundation for optimizing thermal processing technology, controlling the microstructure of composites and improving the mechanical properties. However, to date, there have been few studies on the hot deformation characteristics of nano-SiCp/Al-Si composites fabricated via powder metallurgy.

In this paper, isothermal compression tests were conducted on nano-SiCp/Al-Si composites at 470–530 °C and strain rates of 0.01–5 s^−1^, to analyze the influence of deformation conditions on the hot deformation behavior of the nano-SiCp/Al-Si composite, and a strain-compensation constitutive equation, developed with the Arrhenius equation and Z parameter, is proposed. The purpose of this work was to comprehensively understand the hot deformation behavior of nano-SiCp/Al-Si composites, and therefore provide reference for the secondary processing technology and industrial application of nano-SiCp/Al-Si composites.

## 2. Materials and Methods

**Figure 1:**
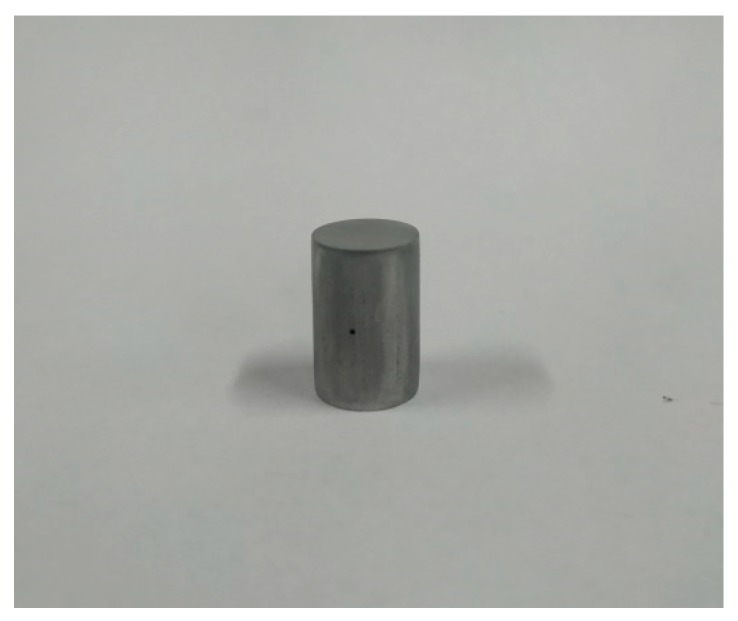
Specimen for thermal compression test.

The 2 vol % nano-SiCp/Al-Si composites were prepared via powder metallurgy by mixing Al-Si powder with a median diameter of 8 μm, and SiC particles with a median diameter of 80 nm. Table 1 shows the chemical composition of the original Al-Si powder. The powders were mixed by a high-energy ball milling method using the QM-BP planetary ball mill (Nanjing nanda instrument plant, Nanjing, China) at a ball milling speed of 150 r/min for 20 h. The mixture was then pressed using a YD32 four-pillar hydraulic machine (Xuzhou dayi heavy forging technology Co., Ltd., Xuzhou, China) at a pressure of 500 MPa for 45 min, and the samples were sintered in a KSS-1200 tube furnace (Luoyang luwei kiln Co., Ltd., Luoyang, China) at 550 °C for 4h. The heating rate was 3 °C/min. The sintered body was hot extruded using an XJ-500 extruder (Wuxi yuanchang machinery manufacturing Co., Ltd., Wuxi, China) at 480 °C with an extrusion speed of 1 mm/s. Then the nano-SiCp/Al-Si composite was annealed in an SX22-510 resistance furnace (Henan offida instrument equipment Co., Ltd., Zhenzhou, China) at 300 °C for 2 h and then cooled to room temperature in the furnace. As shown in Figure 1, the nano-SiCp/Al-Si composites were machined into small cylindrical samples with a diameter of 10 mm and a height of 15 mm by wire cutting, and a 0.5 × 2 mm hole for installing a thermocouple was drilled into the side of the sample.

**Figure 2:**
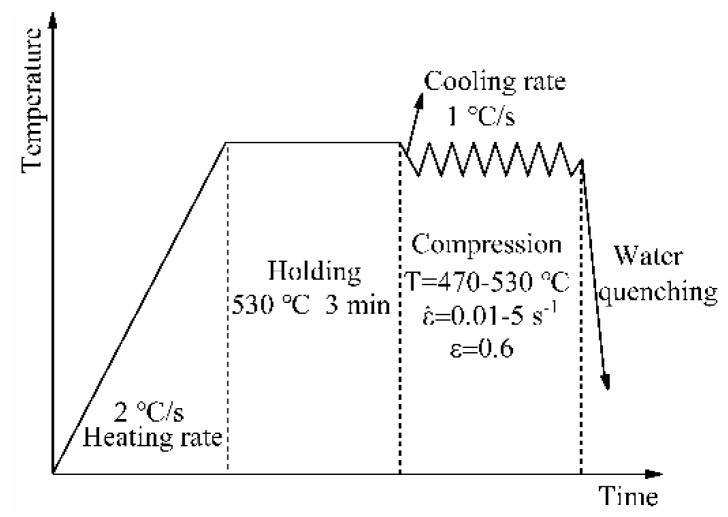
Process diagram of the thermal compression test.

Subsequently, the thermal compression test was conducted on the nano-SiCp/Al-Si composites using a Gleeble-1500D thermal simulator (Dynamic Systems Inc., New York, USA) at 470, 490, 510, and 530 °C, and strain rates of 0.01, 0.1, 1, and 5 s^−1^. To obtain more accurate data, the two ends of the sample were ground before the test, and a mixture of graphite and engine oil was evenly applied to both ends of the sample. All samples were heated at a heating rate of 2 °C/s to 530 °C for 3 min to ensure uniform temperature distribution of the samples. Then the samples were cooled to deformation temperature at a cooling rate of 1 °C/s. After that, the unidirectional thermal compression experiment was conducted at strain rates of 0.01, 0.1, 1, and 5 s^−1^, respectively. The compression stopped when the true strain of the thermal compression sample reached about 0.6. Figure 2 shows the process diagram of the thermal compression test.

## 3. Results and Discussion

### 3.1. Hot Deformation Behavior

**Figure 3:**
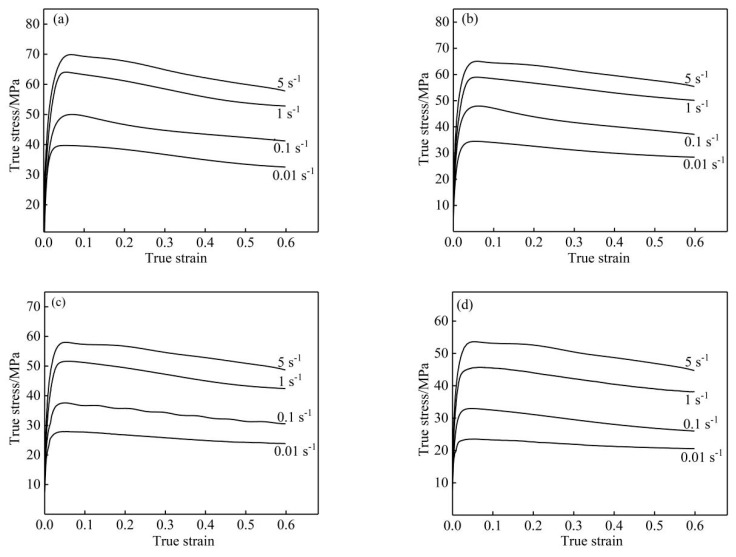
Stress–strain diagrams of nano-SiCp/Al-Si composites: (**a**) 470 °C; (**b**) 490 °C; (**c**) 510 °C; (**d**) 530 °C.

Figure 3 shows the flow curves of nano-SiCp/Al-Si composites obtained through isothermal hot compression experiments at 470–530 °C and strain rates of 0.1–5 s^−1^. It can be seen from Figure 3 that the flow stress was significantly affected by the hot deformation conditions. With the increase in strain, the flow stress of nano-SiCp/Al-Si composites rapidly increased to a peak value during the initial stage of deformation due to the work hardening, and then decreased slowly as a result of dynamic recovery and dynamic recrystallization.

**Figure 4:**
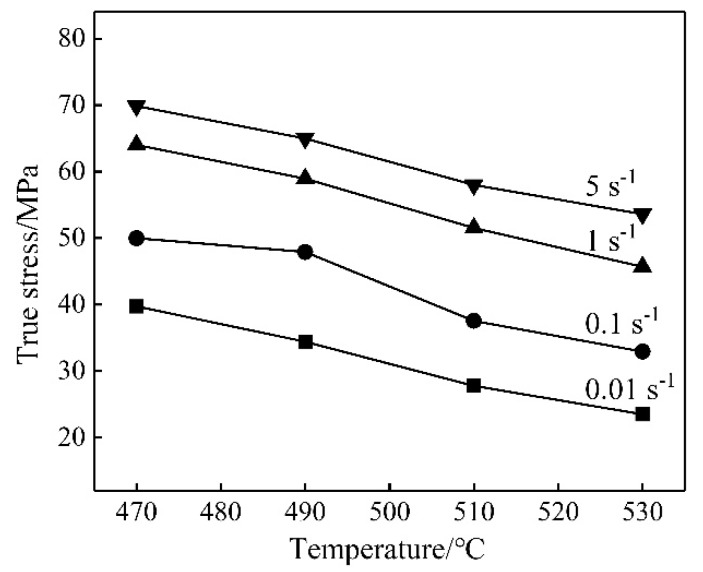
Peak stress of nano-SiCp/Al-Si composites at different conditions.

The variation in peak stress of nano-SiCp/Al-Si composites with hot deformation temperature is shown in Figure 4 for different strain rates. The peak stress of nano-SiCp/Al-Si composites significantly decreased with the increase in the hot deformation temperature at a constant strain rate, indicating that nano-SiCp/Al-Si composites were temperature-sensitive. The peak stress of nano-SiCp/Al-Si composites decreased with the decrease in strain rate at constant deformation temperature, similar to micro-SiC p/Al-Si composites [18]. This behavior may be attributed to the fact that dynamic softening is stronger at high temperature, and there is insufficient time for dynamic softening at a high strain rate [19]. According to the literature [20], the hot deformation activation energy generally increases with the increasing content of the reinforcement. Therefore, the flow stress of the composites might increase with the increasing content of nano-SiC when the volume fraction of nano-SiC particles is low.

### 3.2. Constitutive Equation

The hyperbolic sine function containing the activation energy is commonly used to describe the relationship between flow stress, strain rate, and hot deformation temperature when studying hot deformation behaviors of materials. The expression is [21,22]:

(1) $$\dot{\varepsilon }={A[\sin h(\alpha \sigma )]}^{n}\exp(-Q/RT),\;\mathrm{for}\;\mathrm{all}\;\sigma$$

(2) $$\dot{\varepsilon }=A_{1}\sigma^{n^{\prime }}\exp\left(-Q/\mathrm{RT}\right),\;\sigma \alpha <0.8$$

(3) $$\dot{\varepsilon }=A_{2}\exp\left(\beta \sigma \right)\exp\left(-Q/\mathrm{RT}\right),\;\sigma \alpha >1.2$$

where α, β, A2, A, A1, n′, and n are material constants, and $\beta =\alpha \times n^{\prime }$. $\dot{\varepsilon }$, σ, R, T, and Q represent the strain rate (s^−1^), flow stress (MPa), molar gas constant (8.314 J·mol^−1^·K^−1^), Kelvin temperature (K), and activation energy (kJ/mol), respectively.

Supposing that Q is a constant in a certain deformation temperature range, the natural logarithms of both ends of Equations (1)–(3) can be obtained:

(4) $$\ln\dot{\varepsilon }=\ln A+n\ln[\sin h(\alpha \sigma )]-Q/RT$$

(5) $$\ln\dot{\varepsilon }=\ln A_{1}+n^{\prime }\ln\sigma -Q/RT$$

(6) $$\ln\dot{\varepsilon }=\ln A_{2}+\beta \sigma -Q/RT$$

**Figure 5:**
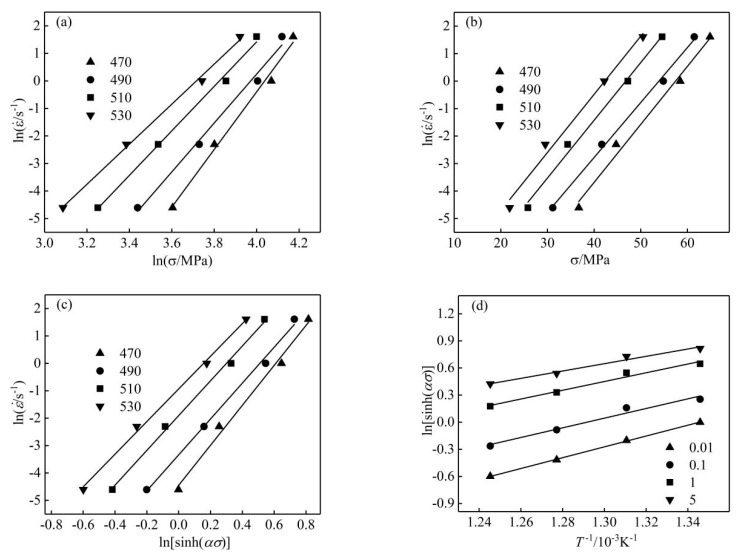
Scatter diagrams and linear regression fits: (**a**) ln$\dot{\varepsilon }$-lnσ; (**b**) ln$\dot{\varepsilon }$-σ; (**c**) ln$\dot{\varepsilon }$-ln[sinh(ασ)]; (**d**) ln[sinh(ασ)]-1/T.

The flow stress at the strain of 0.3 was used to draw the scatter diagrams of ln$\dot{\varepsilon }$ vs. lnσ and ln$\dot{\varepsilon }$ vs. σ respectively, and the data were linearly fitted, as shown in Figure 5a,b. The linear relationship between ln$\dot{\varepsilon }$, lnσ, and σ is evident. The slopes of the straight lines represent the values of material constants, which were obtained by calculating the average values of the slopes of different linear regression lines. The values of β, n’, and α are 0.2081, 8.681, and 0.024 MPa^−1^, respectively.

Transforming Equation (4), the partial derivatives can be obtained:

(7) $$Q=R{\left\{\frac{\partial \ln[\sin h(\alpha \sigma )]}{\partial \left(1/T\right)}\right\}}_{\dot{\varepsilon }}{\left\{\frac{\partial \ln\dot{\varepsilon }}{\partial \ln\left[\sin h(\alpha \sigma )\right]}\right\}}_{T}$$

where $n={\left\{\frac{\partial \ln\dot{\varepsilon }}{\partial \ln\left[\sin h(\alpha \sigma )\right]}\right\}}_{T}$

Figure 5c,d shows the relationship of ln$\dot{\varepsilon }$ vs. ln[sinh(ασ)] and ln[sinh(ασ)] vs. 1/(T/1000) using linear regression. The values of n and Q/1000nR were determined by calculating the average value of the slopes of the fitted lines. Thus, n = 6.533, Q/1000nR = 5.06, and Q = 273 kJ/mol. The Z parameter was introduced to reflect the relationship between the parameters during hot deformation [23]:

(8) $$Z=\dot{\varepsilon }\exp(Q/RT)=A{\left[\sin h\left(\alpha \sigma \right)\right]}^{n}$$

(9) $$\ln Z=\ln\dot{\varepsilon }+Q/RT=\ln A+\mathrm{nln}\left[\sin h\left(\alpha \sigma \right)\right]$$

**Figure 6:**
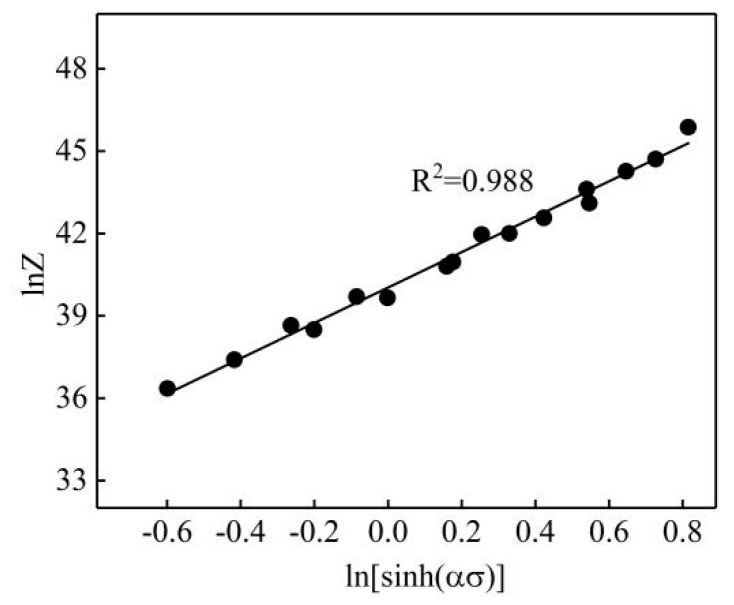
Linear relationship fit of ln[sinh(ασ)]-lnZ.

The corresponding lnZ value was calculated by substituting the deformation condition parameter into Equation (9), and a linear regression was applied to the scatter diagram of lnZ vs. ln[sinh(ασ)]. The result is shown in Figure 6. A = e^40.029^ can be obtained from the intercept on the lnZ axis in the figure.

Substituting material constants such as A, n, Q, and α into Equation (1), the hot deformation constitutive equation of nano-SiC/Al-Si composites with the true strain of 0.3 can be obtained as shown in Equation (10):

(10) $$\dot{\varepsilon }=2.42\times 10^{17}{\left[\sin h\left(0.024\sigma \right)\right]}^{6.533}\exp(-\frac{273000}{\mathrm{RT}})$$

where the units of the quantities 0.024 and 273,440 are 1/MPa and kJ/mol, respectively.

Substitute Equation (8) into Equation (1) to obtain:

(11) $$\sigma =\frac{1}{\alpha }\ln\left\{{\left(\frac{Z}{A}\right)}^{1/n}+{\left[{\left(\frac{Z}{A}\right)}^{2/n}+1\right]}^{0.5}\right\}$$

Therefore, the constitutive equation of nano-SiCp/Al-Si composites with a true strain of 0.3 can be expressed as:

(12) $$\sigma =\frac{1}{0.024}\ln\left\{{\left(\frac{Z}{2.42\times 10^{17}}\right)}^{1/6.533}+{\left[{\left(\frac{Z}{2.42\times 10^{17}}\right)}^{2/6.533}+1\right]}^{0.5}\right\}$$

where $Z=\dot{\varepsilon }\exp(\frac{273000}{\mathrm{RT}})$

### 3.3. Strain Compensation

Many studies have found that the effect of strain cannot be ignored when studying the thermal deformation behavior of materials. Corresponding characteristics are seen in Figure 3; the flow stress varies significantly with strain. However, Equation (10) does not take into account the influence of the true strain. Therefore, it was necessary to enhance the constitutive equation to predict the strain-dependent flow stress.

**Figure 7:**
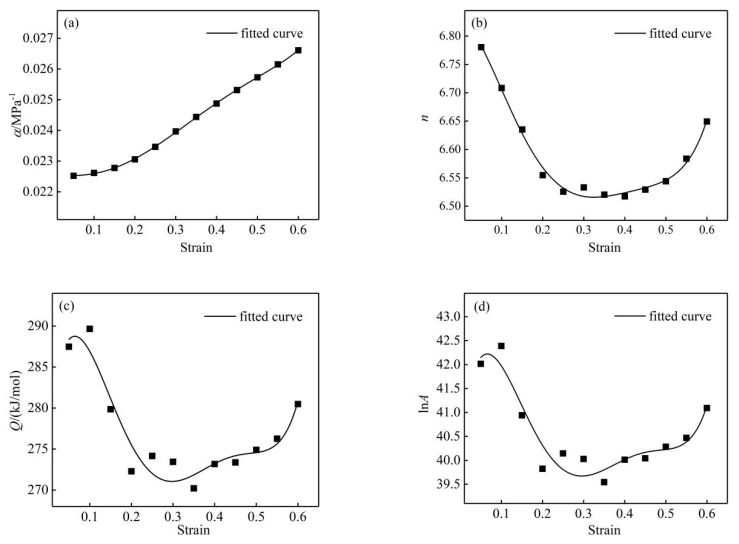
Relationships between true strain and material constants: (**a**) α-ε; (**b**) n-ε; (**c**) Q-ε; (**d**) lnA-ε.

Using the method of calculating the material constants of nano-SiCp/Al-Si composites with a true strain of 0.3 above, the material constants of the composites with a true strain of 0.05, 0.1, 0.15, 0.2, 0.25, 0.3, 0.35, 0.4, 0.45, 0.5, 0.55 and 0.6 were obtained, respectively. The variation in material constant with true strain is shown in Figure 7. The data were accurately fitted using a fifth-degree polynomial, as shown in Equation (13), with coefficients given in Table 2.

(13) $$f\left(\varepsilon \right)=A_{0}+A_{1}\varepsilon +A_{2}\varepsilon^{2}+A_{3}\varepsilon^{3}+A_{4}\varepsilon^{4}+A_{5}\varepsilon^{5}$$

It is generally considered that the activation energy is a physical parameter that characterizes the energy that prevents dislocation movement [12]. The average activation energy (Q) for nano-SiCp/Al-Si composites is 277 kJ/mol, which is larger than the activation energy for self-diffusion of pure aluminum (144 kJ/mol) [16]. According to the literature, the high Q value for nano-SiCp/Al-Si composites can be attributed to the nano-SiC hindering dislocations and the growth of grain boundaries [24,25,26,27]. Senthilkumar et al. [26] compared the deformation activation energy of 5083 aluminum alloy with that of 5083 matrix composite reinforced by nano-TiC and found that the addition of nano-TiC particles increased the activation energy of the material by 30%. Saravanan et al. [27] found that the activation energy of aluminum alloy reinforced by nano-alumina reached 307.23 kJ/mol, which was mainly due to the resistance to dislocation and grain boundary movement caused by nano-alumina particles. Besides, the Q value for nano-SiCp/Al-Si composites was slightly larger than that for Al-Si composites reinforced by micro-SiC (263 kJ/mol) [18], which may be attributed to the nano-SiC retarding the occurrence of the softening mechanism by hindering the growth of grain boundaries or subgrains [14,28].

By substituting the functions of material constants and fitted activation energy into Equation (11), the constitutive equation of nano-SiCp/Al-Si composites under arbitrary true strain is obtained:

(14) $$\sigma (\varepsilon )=\frac{1}{\alpha (\varepsilon )}\ln\left\{{\left(\frac{Z}{A(\varepsilon )}\right)}^{\frac{1}{n(\varepsilon )}}+{\left[{\left(\frac{Z}{A(\varepsilon )}\right)}^{\frac{2}{n(\varepsilon )}}+1\right]}^{0.5}\right\}$$

where $Z=\dot{\varepsilon }\exp(\frac{Q(\varepsilon )}{\mathrm{RT}})$

### 3.4. Strain Compensation Evaluation of Constitutive Equation

**Figure 8:**
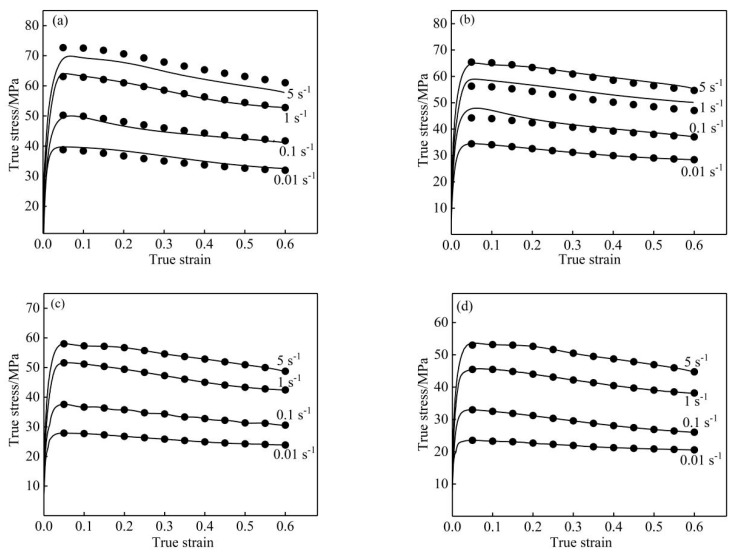
Comparison between experimental and predicted stress of nano-SiCp/Al-Si composites at different deformation conditions: (**a**) 470 °C; (**b**) 490 °C; (**c**) 510 °C; (**d**) 530 °C.

The predicted value of the flow stress corresponding to each strain of the nano-SiCp/Al-Si composites at different temperatures and strain rates was calculated using Equation (14). Figure 8 shows the comparison between experimental and predicted values. The predicted values of the flow stress obtained by the strain-compensation model were close to the experimental values, indicating that the strain compensation constitutive equation established was accurate in predicting the test results.

The deviations between the experimental and predicted values were analyzed statistically using the following formulae to establish the reliability:

(15) $$R=\frac{\sum_{i=1}^{N}\left(E_{i}-\bar{E}\right)\left(P_{i}-\bar{P}\right)}{\sqrt{\sum_{i=1}^{N}{\left(E_{i}-\bar{E}\right)}^{2}\sum_{i=1}^{N}{\left(P_{i}-\bar{P}\right)}^{2}}}$$

(16) $$\mathrm{AARE}=\frac{1}{N}\sum_{i=1}^{N}\left|\frac{E_{i}-P_{i}}{E_{i}}\right|\times 100\%$$

where E_i_ is the experimental stress, $\bar{E}$ is the average value of experimental stress, P_i_ is the predicted stress, $\bar{P}$ is the average value of predicted stress, and N is the number of values used to calculate the error.

**Figure 9:**
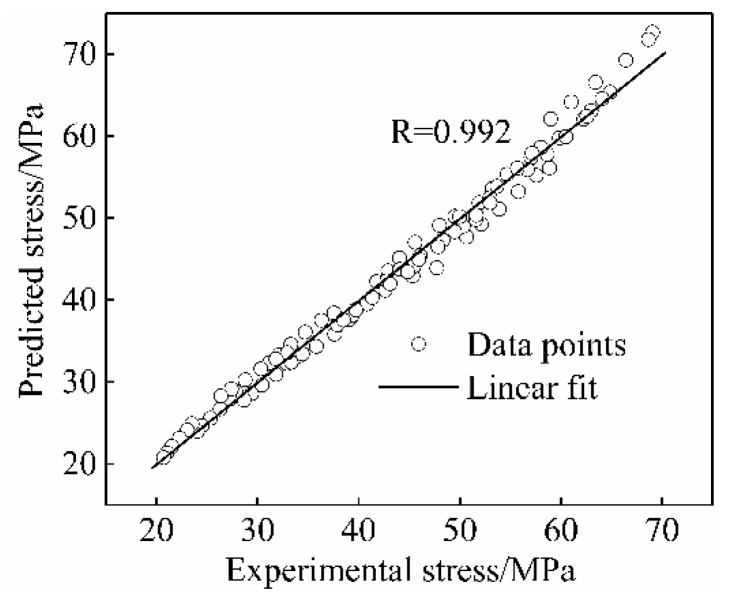
Correlation between experimental and simulated flow stress.

The correlation between the experimental and predicted stress was relatively high, as shown in Figure 9. The experimental and predicted stress corresponding to different strains were substituted into Equations (15) and (16). The average relative error (AARE) was calculated as 2.88% and the correlation coefficient (R) was 0.992, indicating the strain-compensated constitutive equation could predict the hot deformation behavior of nano-SiCp/Al-Si composites accurately. Besides, the difference between model and experimental values may be a result of instability regions that are not accounted for in the strain-compensated constitutive equation [16].

## 4. Conclusions

(1) The flow curves of nano-SiCp/Al-Si composites showed a trend of peaking first and then decreasing gradually with the increase in strain variables, and the stress value was significantly affected by deformation temperature and strain rate.
(2) The average activation energy for the nano-SiCp/Al-Si composites was 277 kJ/mol, which was larger than the activation energy for self-diffusion of pure aluminum.
(3) A strain-compensation constitutive equation was proposed based on isothermal compression test data. The predicted values of the flow stress were consistent with those obtained from experience, indicating that the strain-compensated constitutive equation could predict the hot deformation behavior of nano-SiCp/Al-Si composites accurately.

## Figures and Tables

**Table 1:** Chemical composition of original Al-Si powder (wt %).

Si	Mg	Fe	Al
7	0.3	0.1	Bal.

**Table 2:** Coefficients of α, n, lnA, and Q in Equation (13).

Parameter	A_0_	A_1_	A_2_	A_3_	A_4_	A_5_
α	0.0226	−0.0008	0.0028	0.1299	−0.336	0.2447
n	6.825	−0.1867	−17.22	85.90	−156.3	100.83
Q	278.14	387.89	−4464.85	17287.23	−28326.98	16903.29
lnA	40.56	59.35	−671.91	2591.48	−4237.69	2524.44
